# Activated PI3K-δ syndrome presenting with cervical lymphadenopathy in a pediatric patient: a case report and review of the literature

**DOI:** 10.3389/fimmu.2025.1622764

**Published:** 2025-09-12

**Authors:** Zhiwei Huang, Yaping Wang, Ruoying Wei, Hui Ding, Lian Du, Xiaomei Yang, Fu Li

**Affiliations:** ^1^ Pediatric Hematology and Oncology, Shandong Provincial Maternal and Child Health Care Hospital Affiliated to Qingdao University, Jinan, China; ^2^ Hematology and Oncology, Children’s Hospital Affiliated to Shandong University, Jinan Children’s Hospital, Jinan, Shandong, China

**Keywords:** APDs, PIK3CD, pediatric immunodeficiency, lymphadenopathy, Epstein-Barr virus (EBV)

## Abstract

**Background:**

Activated phosphoinositide 3-kinase delta syndrome (APDS) is a primary immunodeficiency caused by gain-of-function mutations in the PIK3CD gene, leading to dysregulated immune responses. Clinical manifestations include recurrent infections, lymphoproliferation, and increased risk of malignancies. Due to its rarity and variable presentation, APDS is often misdiagnosed or diagnosed late.

**Case presentation:**

We report a case of a 12-year-old girl presenting with persistent cervical lymphadenopathy. Imaging studies revealed extensive lymph node involvement and splenomegaly. PET-CT raised suspicion for lymphoma. Histopathology of the excised lymph node demonstrated Epstein-Barr virus (EBV) latent infection Type III, while whole exome sequencing identified a pathogenic PIK3CD c.3061G>A (p.Glu1021Lys) heterozygous variant. This confirmed the diagnosis of APDS. Targeted therapy with sirolimus was initiated, leading to significant regression of lymphadenopathy and splenomegaly. The patient maintained good clinical status during follow-up.

**Conclusion:**

This case highlights the importance of genetic testing in pediatric patients with lymphoproliferative disease and unusual infection patterns. Early diagnosis of APDS enables personalized, targeted treatment and avoids overtreatment with cytotoxic agents.

## Introduction

Activated phosphoinositide 3-kinase delta syndrome (APDS) is a rare autosomal dominant primary immunodeficiency disorder caused by heterozygous gain-of-function mutations in the PIK3CD gene, which encodes the p110δ catalytic subunit of the class IA phosphoinositide 3-kinase (PI3Kδ) (1). PI3Kδ is primarily expressed in leukocytes and plays a critical role in lymphocyte development, survival, activation, and migration (2). Dysregulation of this signaling pathway leads to excessive immune activation and impaired immune responses, manifesting clinically as recurrent respiratory tract infections, bronchiectasis, lymphadenopathy, and increased risk of lymphoma and other malignancies (1, 2).

APDS typically presents in childhood or adolescence, although milder cases may remain undiagnosed until adulthood. Frequent complications include persistent viral infections especially Epstein-Barr virus, lymphoproliferation, autoimmune cytopenias, and gastrointestinal involvement. Diagnosis is often delayed due to its phenotypic overlap with common variable immunodeficiency (CVID) and other primary immunodeficiencies. Genetic testing for PIK3CD mutations is required for definitive diagnosis (2, 3).

Recent advances in understanding APDS pathogenesis have led to the development of targeted therapies, including mTOR inhibitors (e.g., sirolimus) and PI3Kδ-specific inhibitors, offering new hope for effective and personalized treatment. Early identification and intervention are critical to prevent long-term complications such as structural lung damage, severe infections, and malignancies. This report describes a pediatric case of APDS with prominent cervical lymphadenopathy and splenomegaly, initially mimicking lymphoma, ultimately diagnosed through genetic analysis and successfully managed with sirolimus (3, 4).

## Case description

A 12-year-old girl was admitted to our pediatric hematology and oncology department approximately 10 days after noticing a progressively enlarging mass on the right side of her neck. The mass was initially quail-egg sized, mildly tender to palpation, and was not accompanied by systemic symptoms such as fever, fatigue, night sweats, or weight loss. Her past medical history was notable for the surgical excision of a right breast mass at a local hospital approximately eight months prior, histological documentation was unavailable.

Of particular relevance, the patient experienced significant respiratory illness earlier that year. About three months before presentation, she was reportedly diagnosed with severe pneumonia, fungal infection, and bronchiectasis at a local hospital following a prolonged cough. Although detailed records were not available, her caregivers confirmed that she responded to treatment and was discharged on oral voriconazole. About one month prior to the current admission, she was hospitalized at Jinan Children’s Hospital with a three-day history of hemoptysis. Bronchial artery computed tomography angiography (CTA) revealed bilateral bronchial artery tortuosity, bronchiectasis with active infection, and tracheal diverticulum. She underwent systemic-to-pulmonary collateral embolization and bronchial artery embolization. Concurrent antimicrobial therapy with linezolid and voriconazole led to clinical improvement.

On physical examination, she appeared well-nourished with stable vital signs. Local examination revealed a firm, moderately mobile mass in the right submandibular region, approximately 3.5 × 3.5 cm in size, with mild tenderness and no overlying skin changes. The oral cavity and oropharynx appeared normal. Cardiopulmonary examination was unremarkable. Abdominal palpation revealed splenomegaly, with the spleen tip extending 5 cm below the left costal margin. There was no hepatomegaly or ascites. Additionally, the patient had no history of vaccine side effects.

### Laboratory testing

Initial laboratory evaluation revealed mild leukopenia (WBC 3.2×10^9^/L), reduced lymphocyte count (0.86×10^9^/L), and borderline low neutrophil count (1.8×10^9^/L), along with mild thrombocytopenia (platelet count 94×10^9^/L) and anemia (Hb 98g/L). Inflammatory markers were mild elevated, with a C-reactive protein (CRP) level of 10.68 mg/L and erythrocyte sedimentation rate (ESR) of 27mm/h. Liver and renal function panels were within normal limits.

Serum lactate dehydrogenase (LDH) was mildly elevated at 297 U/L, and β_2_-microglobulin was also mildly elevated at 4.6mg/L. Serological testing revealed positivity for Epstein-Barr virus capsid antigen (EBVCA)-IgG and negativity for EBVCA-IgM, consistent with past or latent EBV infection.

Flow cytometry revealed the following decreased immune cell subset absolute counts: CD3^+^ T cells (620/uL), CD3^+^+CD4^+^ T cells (349/uL), CD3^+^+CD8^+^ T cells (217/uL), CD3^+^-CD16^+^+ CD56^+^ NK cells (217/uL), CD3^+^-CD19^+^ B cells (147/uL). Immunoglobulin testing showed elevated IgM at 7.190g/L, while IgG (8.070g/L) and IgA (0.956g/L) levels were within normal limits.

### Imaging studies

#### Ultrasound examination

**Figure 1:**
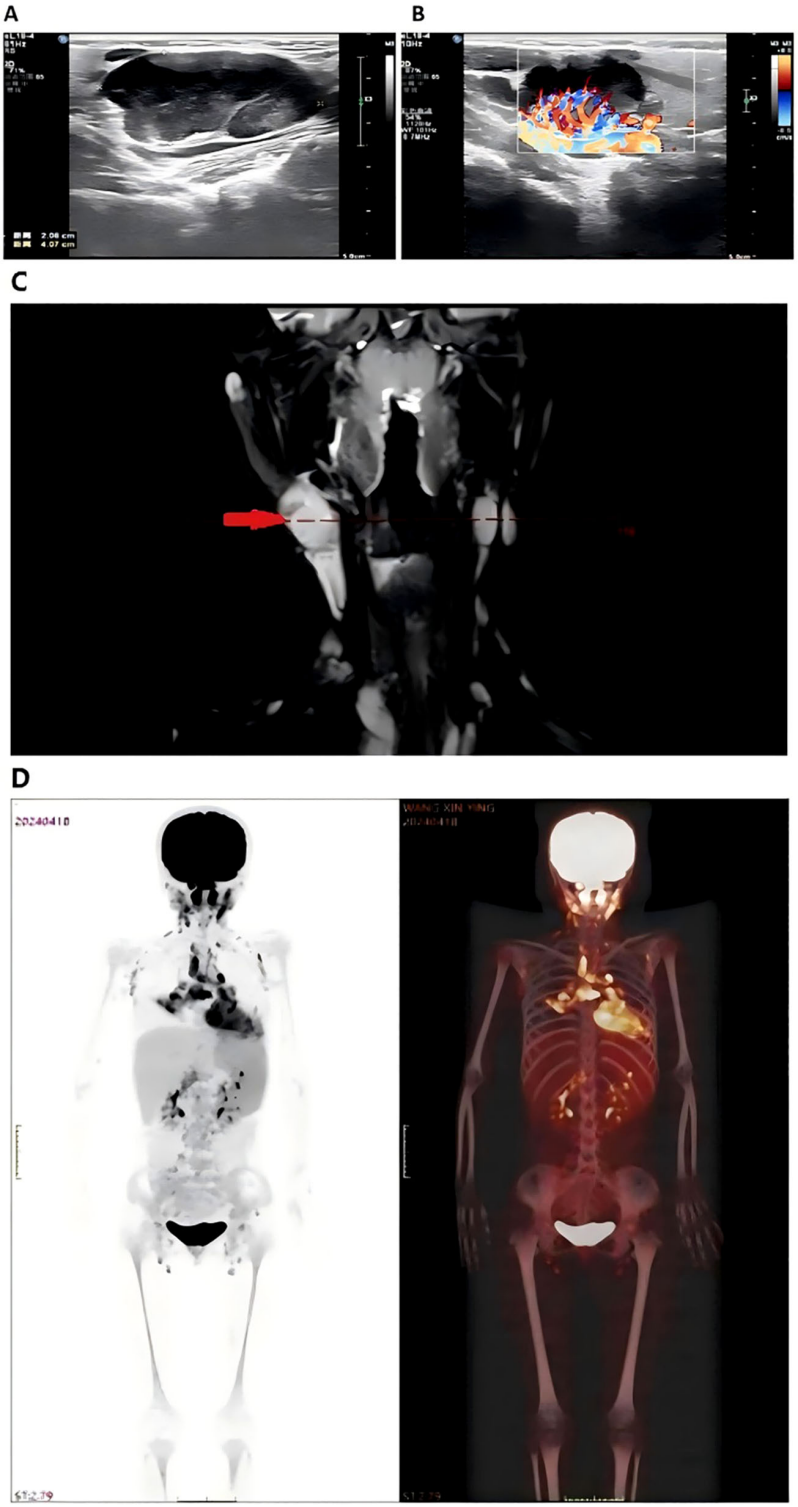
Imaging of bilateral parotid and neck regions showed a hypoechoic mass measuring 4.1×2.8×2.1cm **(A)** surrounded by hypoechoic nodules with abundant blood flow on Doppler imaging **(B)** Neck MRI **(C)** displayed abnormal signals in the right nasopharynx with multiple enlarged lymph nodes, raising suspicion for Hodgkin lymphoma or nasopharyngeal carcinoma. Whole body PET-CT scan **(D)** revealed increased metabolic activity in neck, mediastinal, and abdominal lymph nodes, consistent with lymph node involvement. Splenomegaly and heightened FDG uptake in the thymus were also noted.

High-resolution ultrasound of the neck and bilateral parotid regions revealed a well-defined hypoechoic mass in the right submandibular region, measuring approximately 4.1×2.8×2.1cm. The lesion demonstrated heterogeneous internal echotexture and was surrounded by multiple smaller hypoechoic nodules. Color doppler flow imaging showed rich vascularization within and around the primary lesion, indicating a hypervascular profile (**Figures 1A, B**). No cystic changes or calcifications were observed. These findings were suggestive of a proliferative lymphoid process.

#### Magnetic resonance imaging

MRI of the head and neck demonstrated abnormal signal intensity involving the right nasopharynx and multiple enlarged cervical lymph nodes, predominantly on the right side. These nodes exhibited T2-weighted hyperintensity and mild post-contrast enhancement. The findings raised suspicion for malignant pathology, including Hodgkin lymphoma, non-Hodgkin lymphoma, or nasopharyngeal carcinoma (**Figure 1C**).

#### 18F-FDG positron emission tomography – computed tomography

PET-CT revealed increased fluorodeoxyglucose (FDG) uptake in bilateral cervical, supraclavicular, mediastinal, mesenteric, and para-aortic lymph nodes. Diffusely increased FDG uptake was also noted in an enlarged spleen, consistent with active lymphoid proliferation. Mild hypermetabolism of the thymus was observed, considered abnormal for the patient’s age (**Figure 1D**). No abnormal uptake was seen in the lungs, liver, or skeletal system.

### Histopathological evaluation

An excisional biopsy of the right submandibular lymph node revealed a polymorphic lymphoid infiltrate with architectural effacement and features consistent with EBV-associated lymphoproliferative disease.

**Figure 2:**
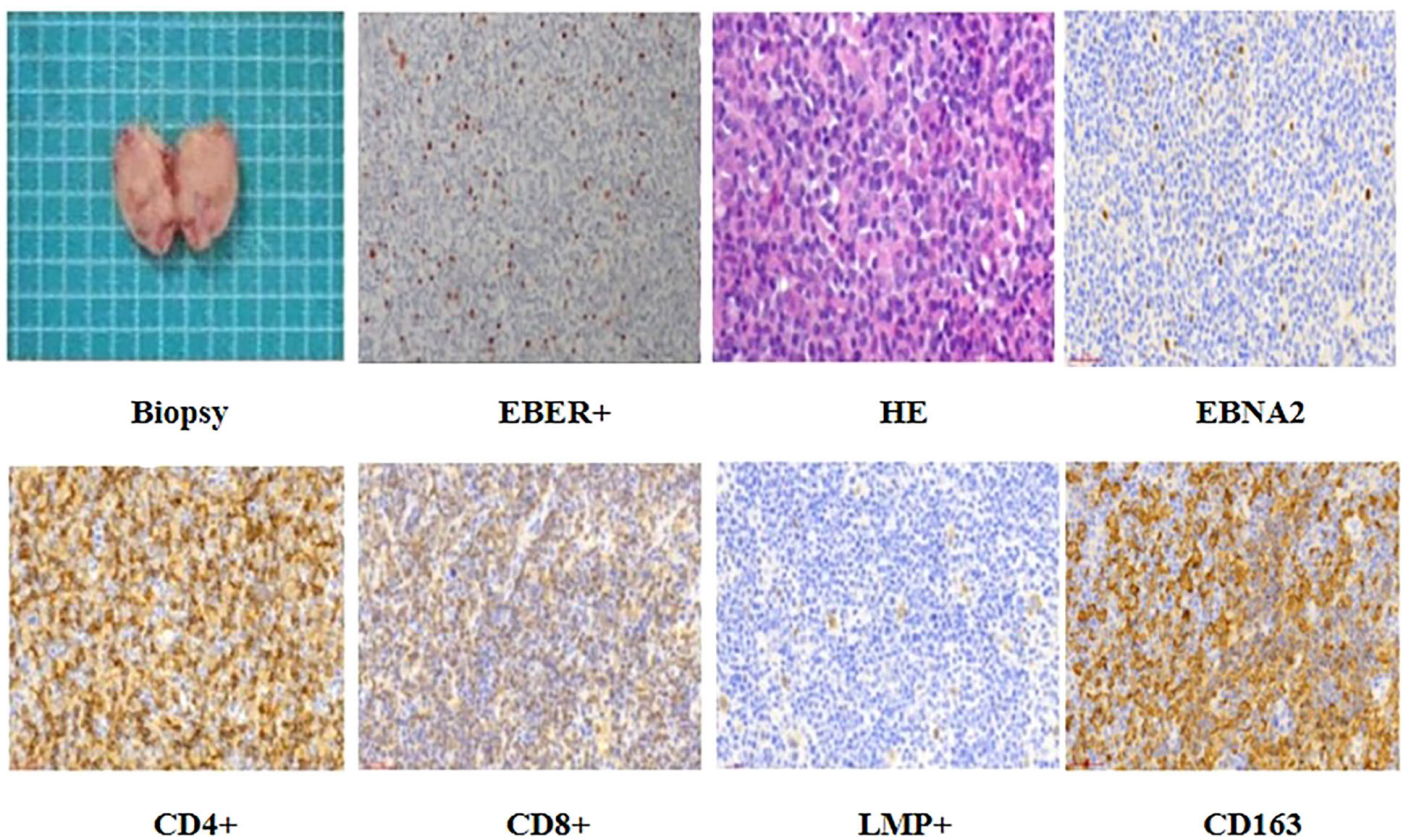
The patient underwent excision of the neck mass, and histopathological examination confirmed a diagnosis of Epstein-Barr virus (EBV) latent infection Type III. Immunohisto chemical analysis showed positivity for CD4, CD8, EBNA2, LMP1, and CD163.

HE staining showed a thickened lymph node capsule, effaced architecture, focal necrosis, and sheets of monomorphic medium-sized lymphoid cells with oval to irregular nuclei, fine chromatin, and inconspicuous nucleoli. Scattered larger cells with prominent nucleoli were also noted. Numerous plasma cells and histiocyte-like cells with evidence of phagocytosis were present in the background, indicating active immune response (**Figure 2**).

Immunohistochemical staining demonstrated abundant CD4^+^ and CD8^+^ T lymphocytes, scattered CD20^+^/CD19^+^ B cells. EBV latency Type III was confirmed by EBNA2, LMP1, and scattered EBER positivity (*in situ* hybridization). CD163 showed focal positivity (**Figure 2**). However, flow cytometry was not performed due to tissue processing limitations.

PCR analysis showed no clonal immunoglobulin (IgH, IgK, Igλ) or T-cell receptor (TCR) gene rearrangements.

### Genetic analysis

Given the patient’s clinical presentation-early-onset lymphoproliferation, EBV-associated pathology, splenomegaly, recurrent infections, and a history of bronchiectasis-a hereditary immunodeficiency was strongly suspected. As part of the diagnostic workup, whole-exome sequencing (WES) was performed to evaluate for monogenic immune dysregulation syndromes.

**Figure 3:**
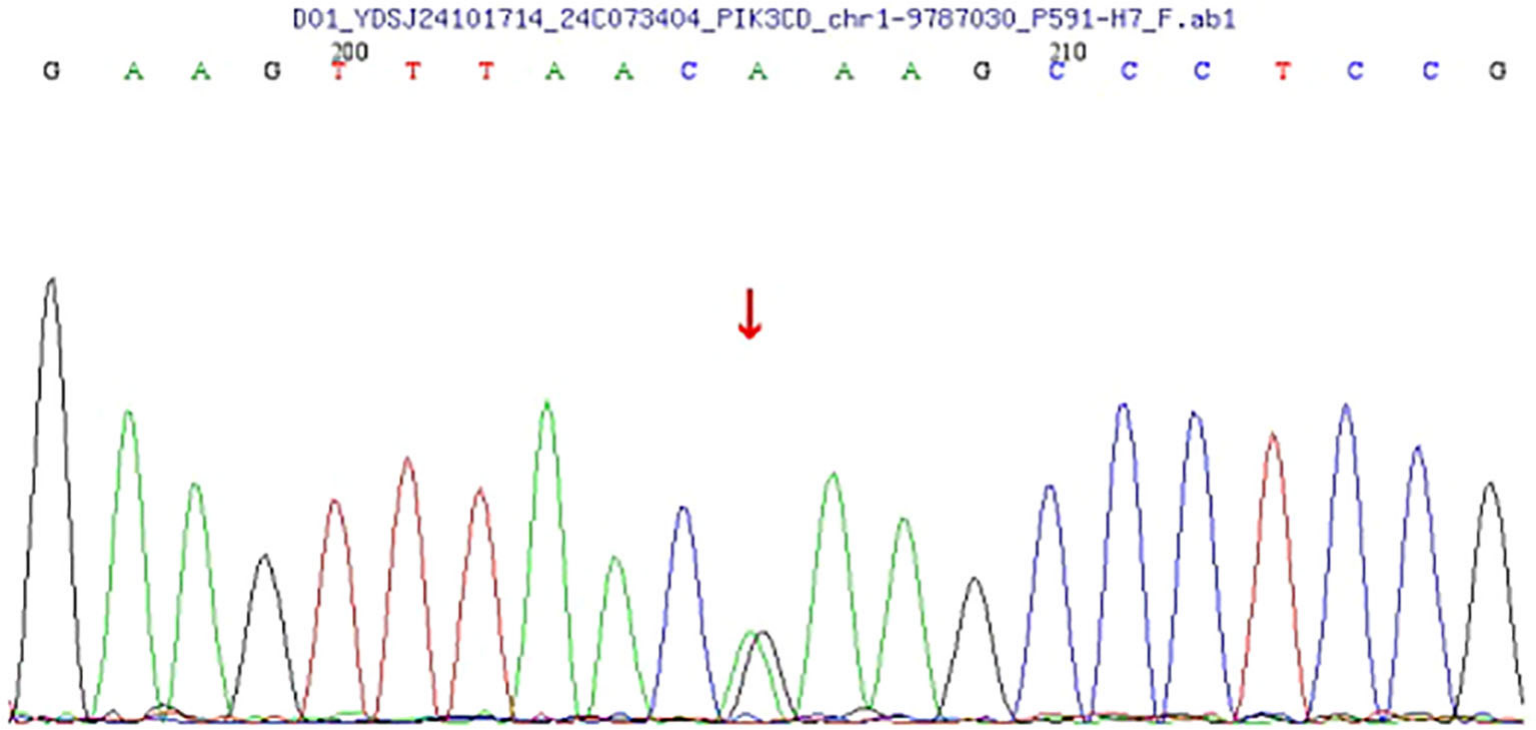
The analysis reveals a heterozygous mutation in the PIK3CD gene at nucleotide 3061 (c.3061G>A), resulting in an amino acid change from glutamate to lysine at position 1021 (p.Glu1021Lys). This mutation was identified through next-generation sequencing (NGS) and verified by Sanger sequencing. This pathogenic mutation may have clinical implications, as the PIK3CD gene is involved in critical signaling pathways affecting immune responses.

Genomic DNA extracted from peripheral blood was analyzed using high-throughput next-generation sequencing (NGS) targeting over 20,000 coding genes. The data were aligned to the human reference genome (GRCh37/hg19), and variant calling was conducted using standard bioinformatics pipelines. The analysis identified a heterozygous missense mutation in the *PIK3CD* gene, specifically: c.3061G > A, resulting in a p. (Glu1021Lys) substitution (**Figure 3**). This mutation lies within the kinase domain of the *PIK3CD* gene, which encodes the p110δ catalytic subunit of phosphoinositide 3-kinase delta (PI3Kδ), a molecule critical for B and T cell function. The Glu1021Lys substitution is a known pathogenic, gain-of-function variant that leads to constitutive activation of the PI3K-AKT-mTOR signaling pathway, driving lymphocyte hyperactivation, impaired memory B-cell development, and heightened susceptibility to infections and lymphoid hyperplasia. This specific variant has been previously reported in multiple cohorts of patients with Activated PI3K-δ Syndrome (APDS) Type 1 and is considered a diagnostic hallmark of the disorder.

To validate the NGS findings, Sanger sequencing was performed and confirmed the presence of the c.3061G >A mutation in a heterozygous state. No additional pathogenic variants were detected in genes commonly associated with primary immunodeficiency or lymphoproliferative syndromes.

Given the autosomal dominant inheritance pattern of APDS, genetic counseling was recommended, and testing of first-degree relatives was proposed to evaluate potential familial transmission. However, at the time of reporting, parental testing was still pending. The identification of this mutation established a molecular diagnosis of APDS1 and directly influenced clinical management, enabling the initiation of targeted therapy with sirolimus, an mTOR pathway inhibitor, aimed at mitigating immune dysregulation and lymphoproliferation.

### Treatment and follow-up

Following genetic confirmation of APDS, the patient was started on sirolimus therapy and antimicrobial prophylaxis. Over the following months, she demonstrated steady clinical improvement, including resolution of lymphadenopathy and splenomegaly (**Supplementary Figure S2**), normalization of blood counts (**Table 1**, **Supplementary Figure S1**), and overall improvement in physical well-being, such as increased appetite, energy, and weight gain.

**Table 1:** Serial complete blood counts (CBC) were monitored from symptom onset.

Time Points from symptom onset	WBC (×10^9^/L)	Neutrophil counts (×10^9^/L)	Lymphocyte counts (×10^9^/L)	Hemoglobin (g/L)	Platelets (×10^9^/L)
Days 10	3.20 (low)	1.80 (normal)	0.86 (low)	98 (low)	94 (low)
Days 56	2.58 (low)	0.88 (low)	1.47 (low)	87 (low)	56 (very low)
Days 110	4.72 (normal)	2.70 (normal)	1.50 (normal)	112 (normal)	136 (normal)
Days 133	4.28 (low)	2.42 (normal)	1.19 (low)	119 (normal)	127 (normal)

Reference ranges: WBC (4.3 - 11.3 ×10^9^/L); Neutrophil Counts (1.6 - 7.8 ×10^9^/L); Lymphocyte Counts (1.5 - 4.6 ×10^9^/L); Hemoglobin (Hb) (118 – 156 g/L); Platelets (150 - 407 ×10^9^/L).

At the most recent follow-up, she remains clinically stable on therapy, without adverse effects. This caregiver perspective highlights the tangible quality-of-life benefits achieved through timely diagnosis and targeted therapy.

## Discussion

Activated PI3K-δ Syndrome (APDS) presents considerable diagnostic and therapeutic challenges due to its overlapping clinical features with other primary immunodeficiencies and lymphoproliferative disorders (2). APDS is caused by gain-of-function mutations in the *PIK3CD* gene, resulting in constitutive activation of the PI3K-AKT-mTOR signaling pathway. This dysregulation disrupts immune homeostasis, leading to a paradoxical state of immunodeficiency coupled with immune hyperactivation, manifesting as recurrent infections, chronic lymphadenopathy, autoimmunity, and an increased risk of malignancies (5, 6).

Our patient exhibited persistent cervical lymphadenopathy and splenomegaly, clinical features that initially raised suspicion for lymphoma—particularly Hodgkin lymphoma or EBV-associated B-cell lymphoma—both recognized complications in APDS (2, 7). The detection of EBV latent infection Type III in lymphoid tissue, commonly seen in post-transplant lymphoproliferative disorders and immunodeficiency-associated cancers, further complicated the clinical assessment and underscored the necessity of precise molecular diagnosis.

This case underscores the critical importance of genetic testing in pediatric patients presenting with atypical or persistent lymphoproliferative disease. Identification of the *PIK3CD* c.3061G>A (p.Glu1021Lys) mutation confirmed the diagnosis of APDS1, obviating empiric cytotoxic chemotherapy and facilitating timely initiation of targeted therapy (2, 8, 9).

Sirolimus, an mTOR inhibitor, has become a cornerstone in APDS management due to its ability to counteract downstream effects of PI3Kδ hyperactivation (7, 10). In our patient, sirolimus treatment resulted in rapid and sustained regression of lymphadenopathy and splenomegaly, alongside normalization of hematologic parameters. The drug was well tolerated, with no significant adverse events reported during follow-up. These findings are consistent with prior studies demonstrating that sirolimus effectively reduces lymphoproliferation, improves immune function, and decreases infection frequency in APDS patients.

Effective management of APDS necessitates a multidisciplinary approach involving pediatric immunologists, hematologists/oncologists, infectious disease specialists, and genetic counselors to address immune dysfunction, monitor for malignancy, and prevent infectious complications (11, 12). Regular monitoring of sirolimus trough levels is vital to optimize therapeutic efficacy while minimizing toxicity.

This case also highlights the need to consider APDS in the differential diagnosis of children presenting with EBV-driven lymphoproliferation or bronchiectasis accompanied by systemic lymphadenopathy (13). APDS may mimic conditions such as common variable immunodeficiency (CVID), EBV-related lymphadenitis, or malignancies. Delayed diagnosis risks unnecessary cytotoxic treatments, heightened infection susceptibility, and poorer long-term outcomes.

This case provides several unique clinical and diagnostic insights that advance the understanding and management of APDS. Notably, the presence of EBV latent infection Type III within lymphoid tissue posed a significant diagnostic challenge, as this form of EBV latency is typically associated with post-transplant lymphoproliferative disorders and immunodeficiency-related malignancies, yet it is less commonly reported in APDS patients. This highlights the complexity of distinguishing benign lymphoproliferation from malignant transformation in the context of EBV-driven disease, underscoring the indispensability of comprehensive molecular and genetic evaluation to avoid misdiagnosis and overtreatment.

Therapeutically, the successful use of sirolimus in controlling both lymphoproliferation and EBV-associated immune dysregulation emphasizes its dual role - not only as an immunomodulatory agent but also as a targeted inhibitor that can mitigate viral-driven lymphoid expansion. The case underscores the challenges in balancing immunosuppression to control lymphoproliferation while preserving sufficient immune competence to prevent EBV reactivation or other opportunistic infections.

Moreover, this report underscores the importance of vigilant longitudinal monitoring for EBV viral load and immune parameters during sirolimus therapy, as viral dynamics in APDS may differ from other immunodeficiencies, potentially necessitating tailored antiviral or adjunctive immunotherapies. These insights collectively enrich the existing literature by illustrating the nuanced interplay between genetic immune dysregulation, viral oncogenesis, and targeted molecular treatment in APDS.

With the increasing accessibility of next-generation sequencing, early and accurate diagnosis of primary immunodeficiencies like APDS is increasingly attainable. Emerging therapies such as PI3Kδ-specific inhibitors (e.g., leniolisib) offer promise for more precise targeted treatment and are currently under clinical investigation (14, 15).

The patient’s early-onset bronchiectasis aligns with previous reports describing recurrent severe pulmonary infections in APDS, which can cause progressive airway damage. Although objective documentation was limited, the history of multiple serious pulmonary infections and requirement for interventional therapies emphasize the importance of considering underlying immunodeficiency in children with early structural lung disease. This case reinforces the diagnostic relevance of APDS in pediatric bronchiectasis of unclear etiology, especially when systemic features or recurrent infections are present.

## Conclusion

This case highlights the importance of early genetic testing in pediatric patients with persistent lymphadenopathy and recurrent infections. Genetic confirmation of APDS allowed for targeted therapy with sirolimus, leading to significant clinical improvement without the need for cytotoxic treatments. Sirolimus effectively managed the patient’s symptoms, supporting its role as a cornerstone in APDS treatment. Early diagnosis and personalized therapy are crucial to avoid overtreatment and improve long-term outcomes. This case also underscores the need for a multidisciplinary approach and raises awareness of APDS as a differential diagnosis in similar clinical presentations.

### Limitations

Although this case report provides valuable clinical and genetic insights intoAPDS, it has certain limitations. Considering the patient’s history of severe pneumonia, fungal infection, and bronchiectasis, a complete immunologic screening would have been beneficial. Such an assessment should ideally include both quantitative and qualitative evaluation of the cellular and humoral components of the immune system. This comprehensive immunological profiling could have provided a more thorough characterization of the patient’s immune status, helped to better delineate the underlying immunopathology, and potentially informed more tailored management strategies.

## Data Availability

The datasets presented in this study can be found in online repositories. The names of the repository/repositories and accession number(s) can be found in the article/**Supplementary Material**.

## Supplementary material

Supplementary Figure 1 Trends in Complete Blood Counts and Lymphoid Organ Size Before and After Sirolimus Treatment. Panel **(A)** illustrates laboratory results and imaging findings across the treatment timeline. On Days 10 from symptom onset, baseline blood tests showed leukopenia **(A)**, low neutrophil ratio **(B)**, decreased lymphocyte ratio **(C)**, thrombocytopenia **(D)**, reduced red blood cell count **(E)**, and anemia **(F)**. On Days 110, most hematological parameters had improved and approached normal levels. At the final follow-up on Days 133, all values remained stable within the normal range.

Supplementary Figure 2 Cervical Lymph Node and Spleen Ultrasound: Images from left to right show pre-treatment (Days 10 from symptom onset: **A, E**) and post-treatment on (Days 56: **B, F**), (Days 110: **C, G**), and (Days 133: **D, H**).At baseline, bilateral cervical lymphadenopathy was present, with a prominently enlarged right-sided node. The spleen measured 3.4 cm in thickness and extended 6.8 cm below the left costal margin. Oral sirolimus (0.64 mL twice daily) was initiated on Days 30. Days 56, ultrasound showed reduction in both lymph node and spleen size, despite low blood counts and a subtherapeutic sirolimus level (3.7 ng/mL), prompting a dose increase to 1 mL twice daily. Subsequent follow-ups demonstrated continued spleen reduction to normal size and marked lymph node shrinkage, though a slight increase was noted at the latest visit. On Days 133, the sirolimus level was 4.2 ng/mL. Clinical response remained favorable, and the current dosage was maintained. Panels **(I, J)** show trends in spleen thickness and subcostal extension over time.
