# Supplementary figures and images for: Activated PI3K-δ syndrome presenting with cervical lymphadenopathy in a pediatric patient: a case report and review of the literature
Source: Front Immunol. 2025 Sep 12;16:1622764. doi: 10.3389/fimmu.2025.1622764 (PMC12463851; doi:10.3389/fimmu.2025.1622764)

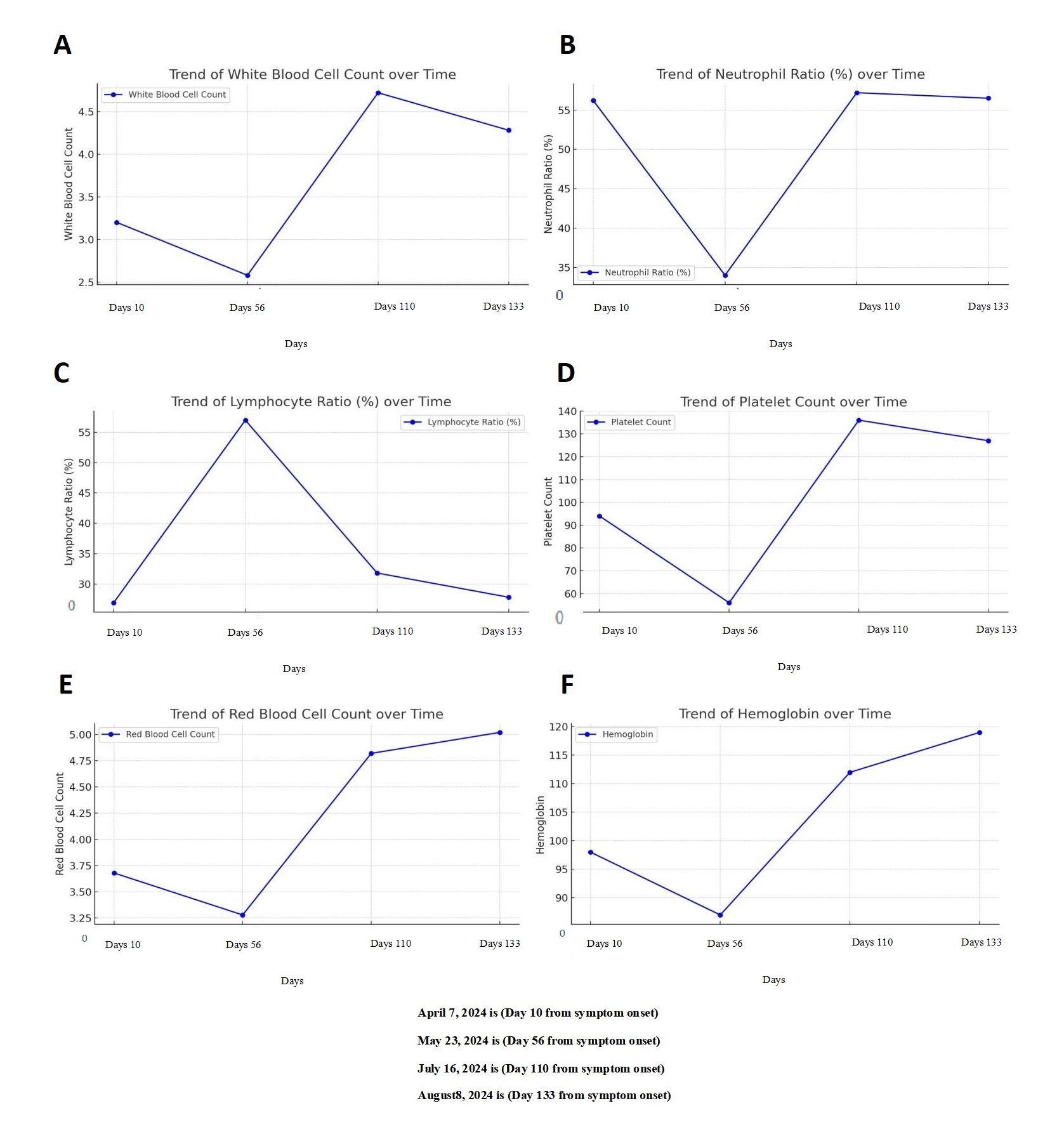

Supplement: Supplementary Figure 1 — Trends in Complete Blood Counts and Lymphoid Organ Size Before and After Sirolimus Treatment. Panel (A) illustrates laboratory results and imaging findings across the treatment timeline. On Days 10 from symptom onset, baseline blood tests showed leukopenia (A), low neutrophil ratio (B), decreased lymphocyte ratio (C), thrombocytopenia (D), reduced red blood cell count (E), and anemia (F). On Days 110, most hematological parameters had improved and approached normal levels. At the final follow-up on Days 133, all values remained stable within the normal range. [file Image1.jpeg]

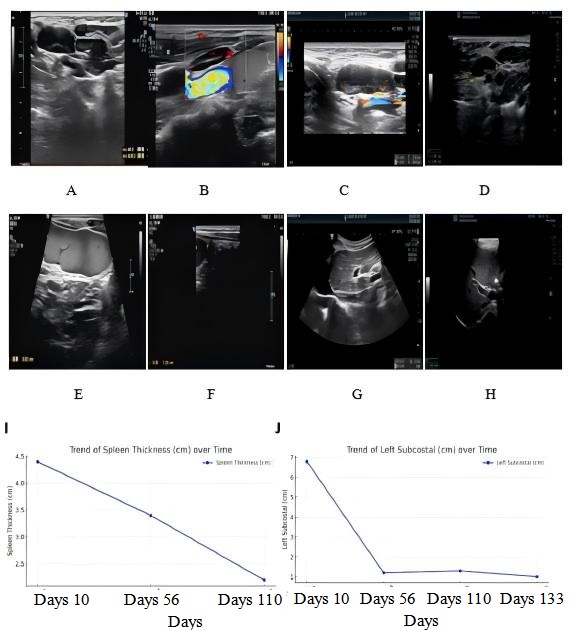

Supplement: Supplementary Figure 2 — Cervical Lymph Node and Spleen Ultrasound: Images from left to right show pre-treatment (Days 10 from symptom onset: A, E) and post-treatment on (Days 56: B, F), (Days 110: C, G), and (Days 133: D, H).At baseline, bilateral cervical lymphadenopathy was present, with a prominently enlarged right-sided node. The spleen measured 3.4 cm in thickness and extended 6.8 cm below the left costal margin. Oral sirolimus (0.64 mL twice daily) was initiated on Days 30. Days 56, ultrasound showed reduction in both lymph node and spleen size, despite low blood counts and a subtherapeutic sirolimus level (3.7 ng/mL), prompting a dose increase to 1 mL twice daily. Subsequent follow-ups demonstrated continued spleen reduction to normal size and marked lymph node shrinkage, though a slight increase was noted at the latest visit. On Days 133, the sirolimus level was 4.2 ng/mL. Clinical response remained favorable, and the current dosage was maintained. Panels (I, J) show trends in spleen thickness and subcostal extension over time. [file Image2.jpeg]
